# Non-contiguous finished genome sequence and description of the gliding bacterium *Flavobacterium seoulense* sp. nov.

**DOI:** 10.1186/1944-3277-9-34

**Published:** 2014-12-29

**Authors:** Su-Kyoung Shin, Heemoon Goo, Yong-Joon Cho, Soonsung Kwon, Dongeun Yong, Hana Yi

**Affiliations:** 1 Department of Public Health Sciences, BK21PLUS Program in Embodiment: Health-Society Interaction, Graduate School, Korea University, Seoul, Republic of Korea; 2 School of Biosystem and Biomedical Science, Korea University, Seoul, Republic of Korea; 3 Chunlab, Inc., Seoul, Republic of Korea; 4 Department of Laboratory Medicine and Research Institute of Bacterial Resistance, Yonsei University College of Medicine, Seoul, Republic of Korea; 5 Korea University Guro Hospital, Korea University, Seoul, Republic of Korea

**Keywords:** *Flavobacterium*, Gliding motility, Aerobic, *Flavobacteriaceae*

## Abstract

*Flavobacterium seoulense* strain EM1321^T^ is the type strain of *Flavobacterium seoulense* sp. nov., a proposed novel species within the genus *Flavobacterium*. This strain is a Gram-reaction-negative, aerobic, rod-shaped bacterium isolated from stream water in Bukhansan National Park, Seoul. This organism is motile by gliding. Here, we describe the features of *Flavobacterium seoulense* EM1321^T^, together with its genome sequence and annotation. The genome comprised 3,792,640 bp, with 3,230 protein-coding genes and 52 RNA genes.

## Introduction

*Flavobacterium* is the type *genus* of the family *Flavobacteriaceae* in the phylum *Bacteroidetes. Flavobacterium* was proposed by Bergey *et al.*[1,2] and the description was emended by Bernardet *et al*. [3]. *Flavobacterium* species have been isolated from various environments, including seawater, freshwater, river sediments, and soil [4-8]. Members of the genus *Flavobacterium* are Gram-negative, rod-shaped, yellow-pigmented, aerobic bacteria. At the time of writing, about 118 *Flavobacterium* species with validly published names have been described [9]; however, the genomes of only 14 type strains in this genus have been sequenced.

*Flavobacterium seoulense* sp. nov. strain EM1321^T^ (= KACC 18114^T^ = JCM 30145^T^) was isolated from stream water in Bukhansan National Park, Seoul, Korea. Here, we present a summary classification and the features of *Flavobacterium seoulense* EM1321^T^ as well as its genome sequence and annotation.

### Classification and features

**Figure 1:**
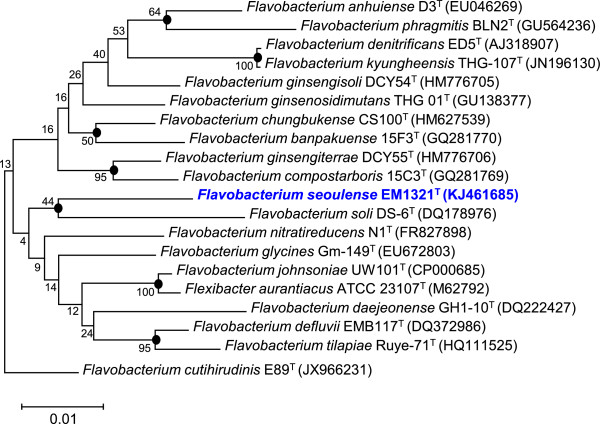
**Phylogenetic tree highlighting the position of *Flavobacterium seoulense *EM 1321**^**T**^**relative to the type strains of other species within the genus *Flavobacterium*.** The strains and their corresponding GenBank accession numbers of 16S rRNA genes are indicated in parentheses. The sequences were aligned using jPHYDIT and the phylogenetic inferences were obtained using neighbour-joining method with MEGA version 5 [13]. The numbers at nodes are the percentage of bootstrap values obtained by 1,000 replicates. Solid circles indicate that the corresponding nodes were also recovered in maximum-likelihood tree. Bar, 0.01 substitutions per nucleotide position.

Based on its 16S rRNA gene phylogeny and phenotypic characteristics, strain EM1321^T^ was classified as a member of the genus *Flavobacterium* (Table 1). Preliminary sequence-based identification using the 16S RNA gene sequences in the EzTaxon database [10] indicated that strain EM1321^T^ was most closely related to *F. granuli* Kw05^T^ (GenBank accession no. AB180738) with a sequence similarity of 96.54%. This value was lower than the 98.7% 16S rRNA gene sequence similarity as a threshold recommended by Stackebrandtia and Ebers [11] to delineate a new species without carrying out DNA-DNA hybridization. Subsequent phylogenetic analysis was performed using the 16S rRNA gene sequences of strain EM1321^T^ and related species. Sequences were aligned according to the bacterial rRNA secondary structure model using the jPHYDIT [12]. Phylogenic trees were constructed using neighbor-joining (NJ) and maximum-likelihood (ML) methods implemented in MEGA version 5 [13]. The resultant tree topologies were evaluated by bootstrap analyses with 1,000 random samplings. Strain EM1321^T^ formed a monophyletic clade together with *Flavobacterium soli*[5] in both the NJ and ML trees; however, the clustering was not supported by the bootstrap analysis (Figure 1). *Flavobacterium nitratireducens*[8] was further recovered as a sister group of the monophyletic clade in the ML tree only. Based on these phylogenetic trees, *F. soli* KACC 17417^T^ and *F. nitratireducens* JCM 17678^T^ were selected as reference strains and were obtained from the corresponding culture collections for comparative study.

**Table 1:** **Classification and general features of *Flavobacterium seoulense *** **EM1321**^**T **^**according to the MICG recommendations**[14]

**MIGS ID**	**Property**	**Term**	**Evidence code**
	Current classification	Domain *Bacteria*	TAS [15]
		Phylum *Bacteroidetes*	TAS [16,17]
		Order *Flavobacteriales*	TAS [17,18]
		Family *Flavobacteriaceae*	TAS [3,19-21]
		Genus *Flavobacterium*	TAS [1-3,22]
		Species *F. seoulense*	IDA
		Strain EM1321^T^	IDA
	Gram stain	Negative	IDA
	Cell shape	Rod-shaped	IDA
	Motility	Gliding	IDA
	Sporulation	Non-sporulating	IDA
	Temperature range	4–35°C	IDA
	Optimum temperature	30°C	IDA
MIGS-6	Habitat	Freshwater	IDA
MIGS-6.3	Salinity	0–4%	IDA
MIGS-22	Oxygen requirement	Aerobic	IDA
	Carbon source	d-glucose, l-arabinose	IDA
MIGS-15	Biotic relationship	Free-living	IDA
MIGS-14	Pathogenicity	Non-pathogenic	NAS
MIGS-4	Geographic location	Seoul, South Korea	IDA
MIGS-5	Sample collection time	September 2013	IDA
MIGS-4.1	Latitude	37°36′52′′N	IDA
MIGS-4.2	Longitude	126°59′19′′E	IDA
	Isolation	Stream water	IDA

Evidence codes - IDA: Inferred from Direct Assay; TAS: Traceable Author Statement (i.e., a direct report exists in the literature); NAS: Non-traceable Author Statement (i.e., not directly observed for the living, isolated sample, but based on a generally accepted property for the species, or anecdotal evidence). These evidence codes are from the Gene Ontology project [23]. If the evidence is IDA, the property was directly observed by one of the authors.

**Figure 2:**
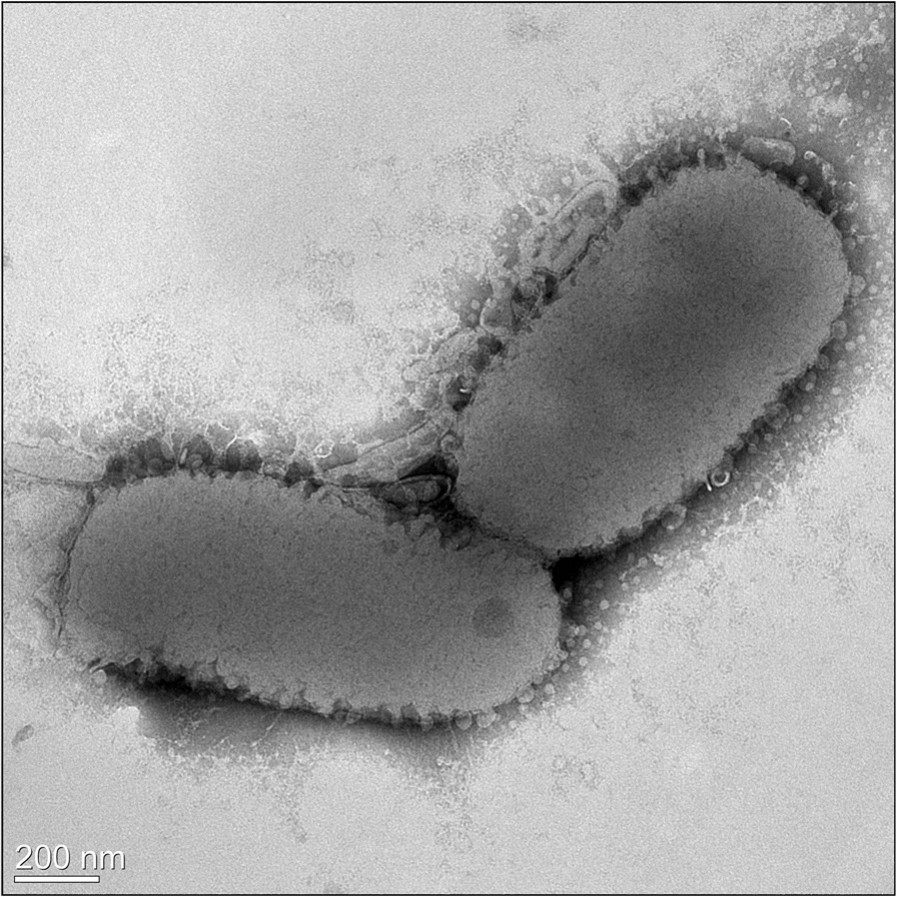
**Transmission electron micrograph of *Flavobacterium seoulense *EM1321**^**T**^**.** Scale bar, 200 nm.

Strain EM1321^T^ was Gram-reaction negative. Cells of strain EM1321^T^ were rod shaped with rounded ends and motile by gliding. The cells were 1.0–1.5 μm × 0.3–0.5 μm in size (Figure 2). No flagellum was observed. The colonies were yellow in color and translucent on R2A agar medium. Growth occurred aerobically at 4–35°C, and optimal growth was observed at 30°C. The cells grew in 0–4% (w/v) NaCl. Strain EM1321^T^ exhibited catalase and oxidase activities. Physiological and biochemical properties were tested by using the API 20NE, API 50CH, and API ZYM systems (BioMérieux). In the API ZYM system, enzyme activity was detected for alkaline phosphatase, esterase (C4), esterase lipase (C8), leucine arylamidase, acid phosphatase, naphthol-AS-BI-phosphohydrolase, *β*-galactosidase, and valine arylamidase (Table 2). No activity was detected for lipase, trypsin, *α*-chymotrypsin, *α*-galactosidase, *β*-glucuronidase, *α*-glucosidase, *N*-acetyl-*β*-glucosaminidase, cystine arylamidase, *α*-mannosidase, and *α*-fucosidase. In the API 20NE system, positive reactions were observed for nitrate reduction and negative reactions were observed for indole production, glucose fermentation, arginine dihydrolase, urease activity, and aesculin and gelatin hydrolysis. The strain assimilated d-glucose and l-arabinose, but not d-mannitol, d-mannose, d-maltose, potassium gluconate, *N*-acetylglucosamine, capric acid, adipic acid, malic acid, trisodium citrate, or phenylacetic acid. Acid was produced from l-arabinose, d-xylose, d-galactose, d-glucose, d-fructose, d-mannose, and d-lactose (API 50CH).

**Table 2:** **Phenotypic characteristics of *Flavobacterium seoulense *** **EM1321**^**T **^**and phylogenetically related *Flavobacterium *** **species**

**Characteristic**	***F. seoulense*** **EM1321**^**T**^	***F. soli*** **KACC 17417**^**T**^	***F. nitratireducens*** **JCM 17678**^**T**^
Cell length (μm)	1.0–1.5	1.0–3.0^a^	1.0–1.5^b^
Oxygen requirement	Aerobic	Aerobic^a^	Aerobic^b^
Gram stain	-	-^a^	-^b^
Salt requirement	0–4%	0–2%^a^	0–1%^b^
Motility	+	+^a^	-^b^
Spore formation	-	-	-
**Production of**			
Alkaline phosphatase	+	+	+
Acid phosphatase	+	+	+
Catalase	+	+	+
Oxidase	+	+	+
Nitrate reductase	+	-	+
Urease	-	-	+
*α*-Galactosidase	-	-	+
*β*-Galactosidase	+	-	-
*β*-Glucuronidase	-	-	-
*α*-Glucosidase	-	-	+
*β*-Glucosidase	-	+*	-
*N*-Acetyl-*β*-glucosaminidase	-	-	+
Indole	-	-	-
Esterase	+	+	+
Esterase lipase	+	+	+
Naphthol-AS-BI-phosphohydrolase	+	+	+
Leucine arylamidase	+	+	+
Cystine arylamidase	-	-	+
Valine arylamidase	+	+*	+
**Utilization of**			
d-glucose	+	-*	+
l-arabinose	+	-*	-
d-mannose	-	-*	+
d-mannitol	-	-	-
d-maltose	-	-*	+
**G + C content (mol%)**	33.25	36.9^a^	36.3^b^
**Habitat**	Freshwater	Soil^a^	Seawater^b^

+: positive result, -: negative result.

^a^Data from Yoon *et al.*[5].

^b^Data from Nupur *et al.*[8].

*Data incongruent with a previous study [5].

**Figure 3:**
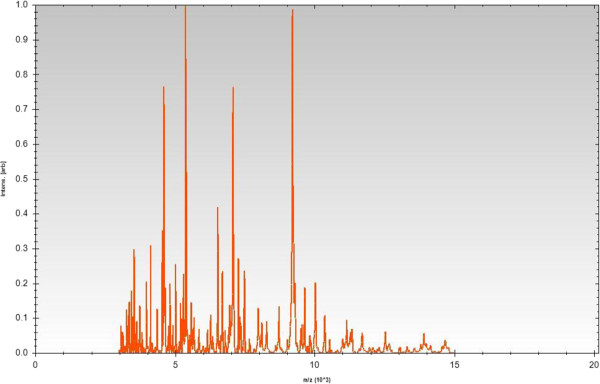
**Reference mass spectrum from *Flavobacterium seoulense *EM1321**^**T**^**.** Spectra from 12 individual colonies were compared and a reference spectrum was generated.

**Figure 4:**
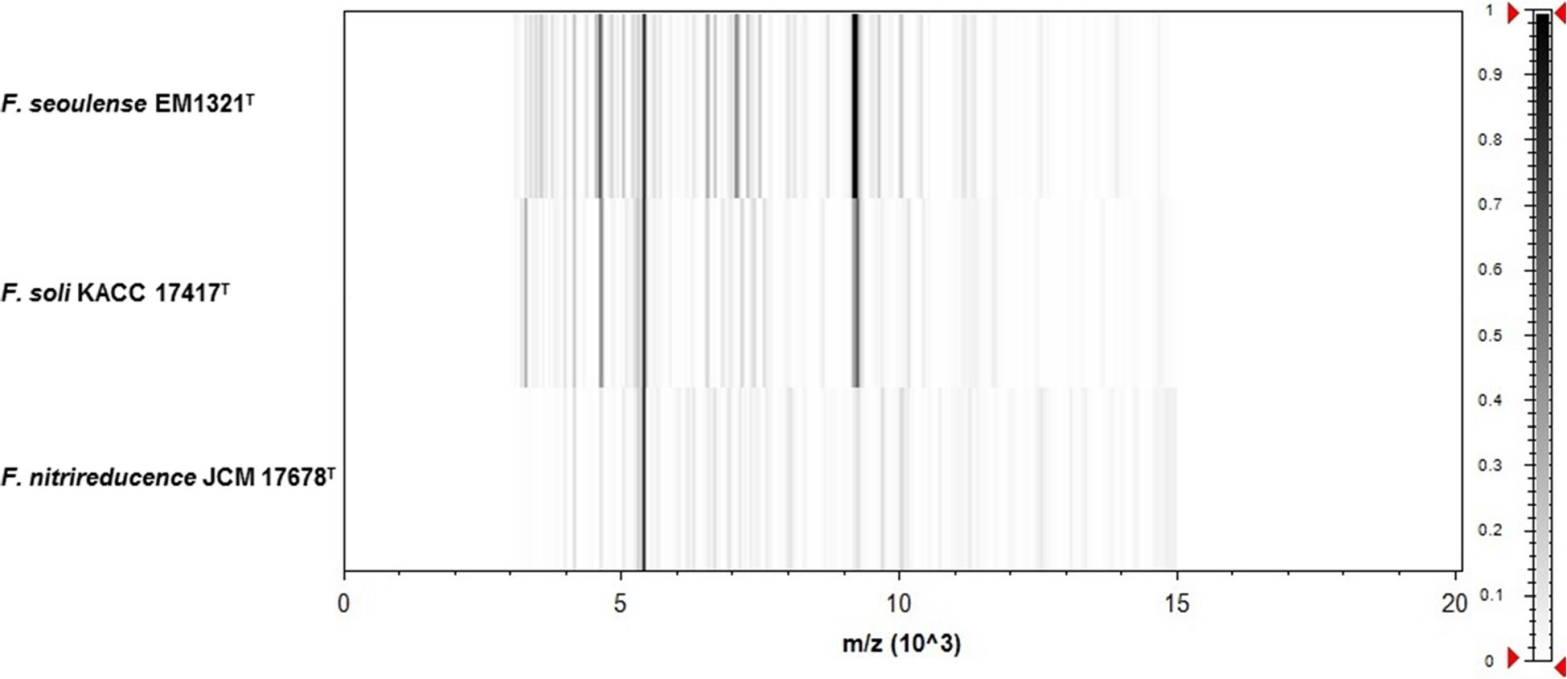
**Gel view comparing the *Flavobacterium seoulense *EM1321**^**T **^**spectrum with those of other members in the genus *Flavobacterium*.** The gel view displays the raw spectra of all loaded spectrum files arranged in a pseudo-gel-like look. The x-axis records the m/z value. Peak intensity is shown as a gray-scale scheme code. The color bar and the right y-axis indicate the relation between the color of a peak and peak intensity in arbitrary units.

Matrix-assisted laser-desorption/ionization time-of-flight (MALDI-TOF) MS protein analysis was carried out as previously described [24]. Deposits were done from 12 isolated colonies for each strain (strain EM1321^T^ and reference strains). Measurements were made with a Microflex spectrometer (Bruker Daltonics, Leipzig, Germany). Spectra were recorded in the positive linear mode for the mass range of 2,000 to 20,000 Da (parameter settings: ion source 1 (IS1), 20 kV; IS2, 18.5 kV; lens, 7 kV). The time of acquisition was between 30 seconds and 1 minute per spot. The twelve EM1321^T^ spectra were imported into the MALDI BioTyper software (version 2.0; Bruker) and analyzed by standard pattern matching (with default parameter settings) against 4,613 bacterial spectra including eight *Flavobacterium* species, used as reference data, in the BioTyper database. For strain EM1321^T^ spectrum (Figure 3), no significant score was obtained, suggesting that our isolate was not a member of the eight known species in the database. Spectrum differences with the two closely related *Flavobacterium* species are shown in Figure 4.

## Genome sequencing information

### Genome project history

*Flavobacterium seoulense* EM1321^T^ was selected for genome sequencing based on its phylogenetic position and its 16S rRNA similarity to other members of the genus *Flavobacterium*. The genome sequence was deposited in GenBank under accession number JNCA00000000.1. A summary of the project and the Minimum Information about a Genome Sequence (MIGS) [14] are shown in Table 3.

**Table 3:** Genome sequencing project information

**MIGS ID**	**Property**	**Term**
MIGS-31	Finishing quality	High-quality draft
MIGS-28	Libraries used	One paired-end Illumina library
MIGS-29	Sequencing platforms	Illumina MiSeq
MIGS-31.2	Fold coverage	166×
MIGS-30	Assemblers	CLCbio CLC Genomics Workbench, version 6.5.1
MIGS-32	Gene calling method	Glimmer 3.0
	Genbank ID	JNCA00000000.1
	Genbank Date of Release	2014/05/27
	BIOPROJECT	PRJNA248341
	Project relevance	Environmental, Biotechnological
MIGS-13	Source Material Identifier	KACC 18114, JCM 30145

### Growth conditions and DNA isolation

*Flavobacterium seoulense* EM1321^T^ was cultured aerobically on R2A agar medium at 30°C. Genomic DNA was extracted using the QIAamp DNA mini kit (Qiagen).

### Genome sequencing and assembly

The genome of strain EM1321^T^ was sequenced at ChunLab, Inc. by using an Illumina Miseq_PE_300 system with 2 × 300 paired-end reads. The Illumina platform provided 166× coverage (for a total of 3,792,640 sequencing reads) of the genome. CLC Genomics Workbench (ver. 6.5.1) was used for sequence assembly and quality assessment. The final draft assembly contained 56 contigs.

### Genome annotation

The genes in the assembled genome were predicted with Rapid Annotation using Subsystem Technology (RAST) server databases [25] and the gene-caller GLIMMER 3.02 [26]. The predicted ORFs were annotated by searching clusters of orthologous groups (COGs) [11] using the SEED database [27]. RNAmmer 1.2 [28] and tRNAscan-SE 1.23 [29] were used to identify rRNA genes and tRNA genes, respectively. CRISPR repeats were examined using CRISPR recognition tool (CRT) [30]. CLgenomics™ 1.06 (ChunLab) was used to visualize the genomic features.

## Genome properties

**Figure 5:**
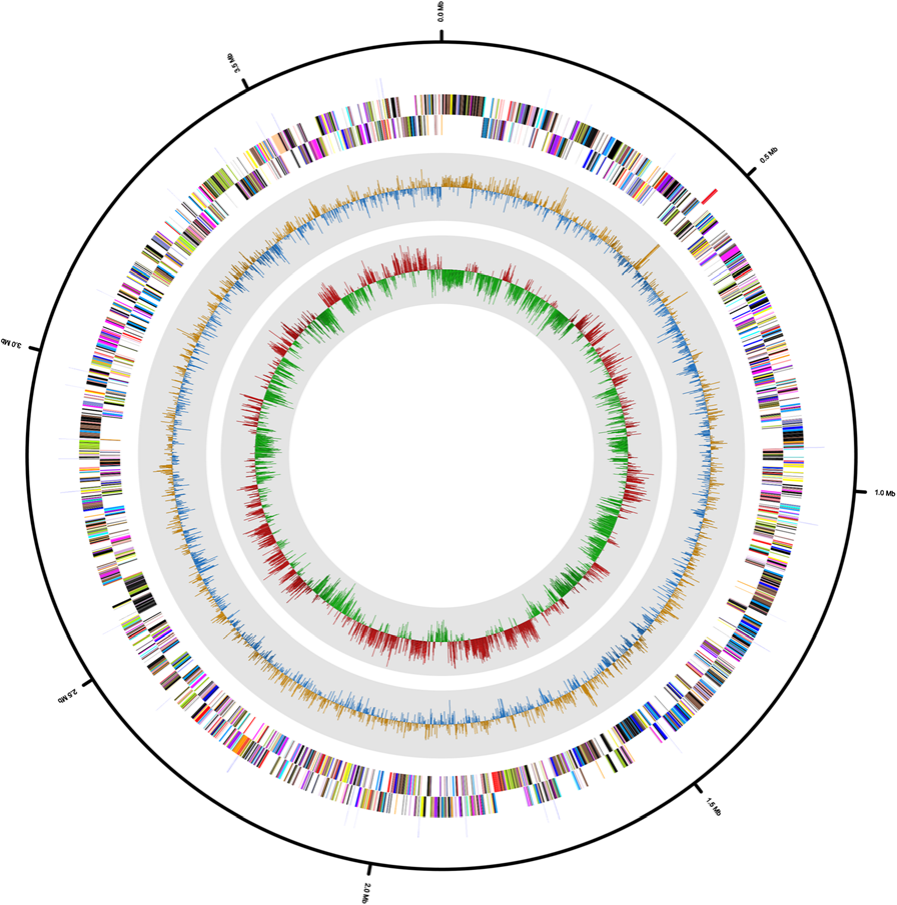
**Graphical circular map of the genome.** Starting from the outmost circle and moving inwards, each ring of the circle contains information on a genome: rRNA/tRNA, genes on the reverse strand (colored according to the COG categories), genes on the forward strand (colored according to the COG categories), GC skew, and GC ratio.

The genome comprised a circular chromosome with a length of 3,792,640 bp and 33.25% G + C content (Figure 5 and Table 4). It is composed of 56 contigs. Of the 3,282 predicted genes, 3,230 were protein-coding genes and 52 were RNA genes (2 rRNA genes and 50 tRNA genes). The sequencing coverage of rRNA operon (673×) indicated that 4 copies of rRNA operons are exist in this genome. The majority of the protein-coding genes (2,054 genes, 62.58%) were assigned putative functions, while the remaining genes were annotated as hypothetical proteins (1,176 genes, 35.83%). The properties of and statistics for the genome are summarized in Table 4. The distribution of genes into COG functional categories is presented in Table 5 and Figure 5.

**Table 4:** Genome statistics

**Attribute**	**Value**	**% of total**^**a**^
Genome size (bp)	3,792,640	100
DNA coding region (bp)	3,386,688	89.30
G + C content (bp)	1,261,070	33.25
Total genes	3,282	100
RNA genes	52	1.58
rRNA operons	4	-
Protein-coding genes	3,230	98.42
Pseudo genes	45	1.37
Genes with function prediction	2,054	62.58
Genes assigned to COGs	2,281	69.50
Genes assigned Pfam domains	1,997	60.85
Genes with signal peptides	119	3.63
Genes with transmembrane helices	682	20.78
CRISPR repeats	0	-

^a^The total is based on either the size of the genome in base pairs or the total number of protein-coding genes in the annotated genome.

**Table 5:** Number of genes associated with the 25 general COG functional categories

**Code**	**Value**	**%**^**a**^	**Description**
J	157	4.86	Translation
A	1	0.03	RNA processing and modification
K	148	4.58	Transcription
L	123	3.81	Replication, recombination, and repair
B	0	0.00	Chromatin structure and dynamics
D	23	0.71	Cell cycle control, mitosis, and meiosis
Y	0	0.00	Nuclear structure
V	40	1.24	Defense mechanisms
T	121	3.75	Signal transduction mechanisms
M	220	6.81	Cell wall/membrane biogenesis
N	18	0.56	Cell motility
Z	0	0.00	Cytoskeleton
W	0	0.00	Extracellular structures
U	45	1.39	Intracellular trafficking and secretion
O	81	2.51	Posttranslational modification, protein turnover, and chaperones
C	122	3.78	Energy production and conversion
G	207	6.41	Carbohydrate transport and metabolism
E	170	5.26	Amino acid transport and metabolism
F	62	1.92	Nucleotide transport and metabolism
H	127	3.93	Coenzyme transport and metabolism
I	93	2.88	Lipid transport and metabolism
P	170	5.26	Inorganic ion transport and metabolism
Q	42	1.30	Secondary metabolites biosynthesis, transport, and catabolism
R	318	9.85	General function prediction only
S	196	6.07	Function unknown
-	949	29.38	Not in COGs

^a^The total is based on the total number of protein coding genes in the annotated genome.

## Conclusions

Based on the results from phylogenetic and phenotypic analyses, we formally propose the creation of the new species *Flavobacterium seoulense* sp. nov. for strain EM1321^T^. The non-contiguous genome sequence of the type strain was determined and described here.

### Description of *Flavobacterium seoulense* sp. nov

*Flavobacterium seoulense* (seo.ul.en’se. N.L. neut. adj., named after Seoul, Korea, the geographical origin of the type strain).

Aerobic, Gram-reaction negative. Cells are rod shaped and motile by gliding. Does not have a flagellum. The colonies are yellow in color and translucent on R2A agar medium. Grows at 4–35°C, with optimum growth at 30°C and in 0–4% (w/v) NaCl. Catalase- and oxidase-positive. Positive for alkaline phosphatase, esterase (C4), esterase lipase (C8), leucine arylamidase, acid phosphatase, naphthol-AS-BI-phosphohydrolase, *β*-galactosidase, and valine arylamidase. Positive for nitrate reduction, but negative for indole production, glucose fermentation, arginine dihydrolase, urease activity, and aesculin and gelatin hydrolysis. Negative for lipase, trypsin, *α*-chymotrypsin, *α*-galactosidase, *β*-glucuronidase, *α*-glucosidase, *β*-glucosidase, *N*-acetyl-*β*-glucosaminidase, or cystine arylamidase activity. This strain assimilated d-glucose and l-arabinose, but not d-mannitol, d-mannose, d-maltose, *N*-acetylglucosamine, potassium gluconate, capric acid, adipic acid, malic acid, trisodium citrate, or phenylacetic acid. Produces acid from l-arabinose, d-xylose, d-galactose, d-glucose, d-fructose, d-mannose, and d-lactose.

The G + C content of the genome is 33.25%. The 16S rRNA and genome sequences are deposited in GenBank under accession numbers KJ461685 and JNCA00000000.1, respectively. The type strain EM1321^T^ (= KACC 18114^T^ = JCM 30145^T^) was isolated from stream water in Bukhansan National Park, Seoul, Korea.

## Competing interests

The authors declare that they have no competing interests.

## Authors’ contributions

SS drafted the manuscript, performed laboratory experiments, and analyzed the data. HG cultured samples and performed the electron micrograph and phylogenetic analysis. YC, SK and DY sequenced, assembled, and annotated the genome. HY organized the study and drafted the manuscript. All authors read and approved the final manuscript.
